# Relief, Recovery, Reform: A Retrospective on the US Policy Responses to the Great Recession

**DOI:** 10.1007/s10272-020-0910-4

**Published:** 2020-07-28

**Authors:** Basak Kus

**Affiliations:** Department of Sociology, Wesleyan University, 238 Church Street, CT 06459-0012 Middletown, USA

## Abstract

The Great Recession presented the US with economic and political challenges that had not been experienced since the time of the Great Depression. This article provides a retrospective on the government’s response to the crisis ten years after the enactment of the Dodd-Frank Act, as the country is heading into its next presidential election in the midst of yet another downturn, this time fueled by the COVID-19 pandemic.

When the Republican candidate for president, Senator John McCain, declared the fundamentals of the American economy to be strong on September 15, 2008, the day Lehman Brothers filed for bankruptcy, his words sounded familiar to students of American political economy. On October 25, 1929, only four days before Black Tuesday, President Herbert Hoover had declared that the fundamentals of American business were "on a sound and prosperous basis" (U.S. Congress, 1929, 5070). In the months that followed, production began to fall drastically and unemployment increased on an unprecedented scale. By October 1931, the number of unemployed had reached nine million, entire neighborhoods in industrial towns had been devastated, and farm and home foreclosures had risen sharply (Fox Piven and Cloward, 1977). The economy, which was believed to be "roaring" in the preceding decade, had plunged into the Great Depression.

The economic crisis of the late 2000s – now referred to as the Great Recession – did not end up being as deep or as long as its predecessor, but it was still severe, as shown in Figure 1. Over the course of the crisis, the unemployment rate increased to 10%, and the GDP fell by over 4%. More than 450 banks failed, and more than 10 million houses were foreclosed (Grusky et al., 2011; Martin and Niedt, 2015). The share of households with negative net worth climbed to 22.5% (Wolf, 2012), and millions of Americans had to postpone their retirement or completely lost the possibility of ever being able to retire.

* An earlier version of this was presented at MaxPo in Paris. I thank those in the audience for their feedback. Many thanks also to Onur Ozgode for reading an earlier draft of this article and giving helpful feedback. All errors are my own.

**Basak Kus**, Wesleyan University, Middletown, CT, USA.

This article provides a retrospective on the government's response to the crisis, as the country is heading into its next presidential election in the midst of yet another downturn, this time fueled by a pandemic. This is, therefore, a particularly good time to think about government intervention in times of crises. The article provides an overview of the relief, recovery and reform measures taken in response to the Great Recession. It highlights the key issues and concerns that remained salient to each aspect of the government's response, and how those issues were understood and tackled by those who were at the helm of policymaking.

## Politics in hard times[1]: From uncertainty to action during the Great Recession

Crises are times of uncertainty, and they often make space for policy change. As Hay elaborates, "the very parameters that previously circumscribed policy options are cast asunder and replaced, and the realm of politically possible, feasible and desirable is correspondingly reconfigured" (Hay, 2001, 197). Before uncertainty turns into action, however, political actors must first decide what the crisis is actually a crisis of, and this tends to be a contested process (Blyth, 2002). An important point of contention in these political deliberations concerns the role of the government. Disagreements abound about the size, substance and tools of government intervention;

1 Subtitle "Politics in hard times" is a hat tip to Peter Gourevitch's book of the same title.

Figure 1
**Great Recession: Economic indicators**

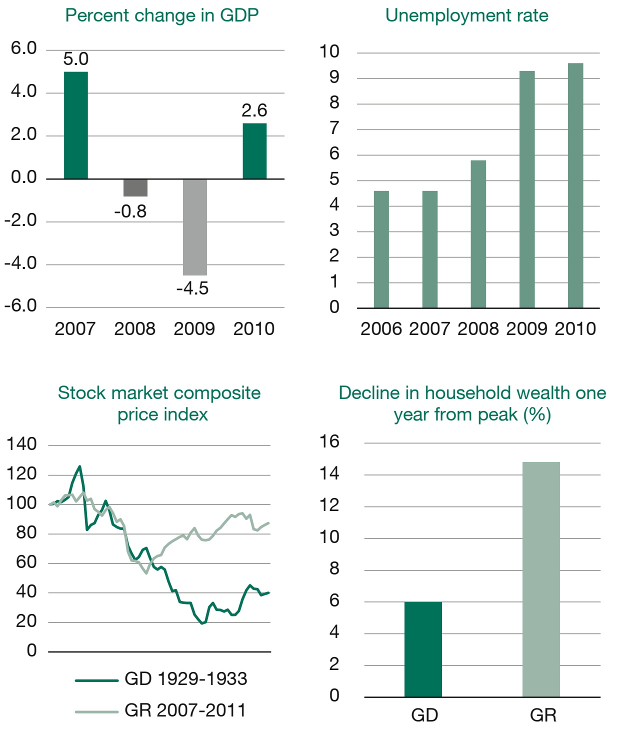

Note: GD: Great Depression; GR: Great Recession.

Sources: GDP data from the Bureau of Economic Analysis (Q1); unemployment data from the Bureau of Labor Statistics; Stock market data from R. Shiller, "U.S. Stock Markets 1871-Present" data set; data on household wealth from Charting the Financial Crisis: US Strategy and Outcomes, joint report by Hutchins Center on Fiscal and Monetary Policy at Brookings and Yale School of Management Program on Financial Stability.

who deserves help and who does not; which trade-offs are acceptable, and which are not. The 2007-2010 economic crisis was no exception in this regard.

The government's initial response was a figure-it-out-as-you-go involving various emergency initiatives aimed at staunching the blood loss in the economy. Decisions had to be made swiftly and with limited foresight. The former Fed Chair Bernanke recalls:

> When I first became chairman of the Federal Reserve in 2006, literally one of the first things I did was I asked the staff to give me the handbook on what you do in the case of a financial crisis. And they provided me a little notebook and the notebook was typed on a manual typewriter in mimeograph and had about four pages in it. It said open the discount window and that was about it…I think Tim Geithner had a similar experience at the New York Fed. We went into one of the most complicated and consequential financial crises in human history with very little in the way of a playbook for thinking about how to address the crisis. (2018)

The Fed played a significant role in this stage, supplying large quantities of reserves to tackle the dislocations in financial markets. Early on in the timeline of the crisis, however, many of the risk-mitigation tools that the Treasury, the Fed and the Federal Deposit Insurance Corporation had at their disposal were aimed at depository institutions. Non-bank financial institutions – although they had become a significant part of the American financial system in the decades preceding the crisis – did not have access to those same tools and this posed a challenge that deeply concerned policymakers (Alvarez and Dudley, 2018). Senior officials who served at the New York Fed at the time recall:

> As we stood on the eve of the financial crisis, the non-bank sector was very fragile. When the housing bubble burst and home prices declined, we knew that the stress on this sector was going to increase, and as it increased there was very little in the tool kit that the regulatory authorities had to respond as circumstances deteriorated. And I think that made it just much more difficult to arrest the downward dynamic and to restore confidence. So, in 2008, when we were faced with the eminent failures of Bear Sterns, Lehman and AIG in short order, policymakers were faced with an important question – how far could they go in stretching the available tools, which were clearly inadequate, to limit contagion and constrain an ever-broadening financial crisis? (Dudley, 2018)

> A systemic crisis calls for a central bank lending to the broad financial system basically temporarily substituting for the breakdown in private lending relationships until the system can stabilize itself. But that absolutely can't happen if the lending facility actually excludes most of the financial system. So, distress at non-banks continued and accelerated. (Mosser, 2018)

It was within these circumstances that the Fed, for the first time since the Great Depression, invoked in March 2008 its emergency authority under section 13(3)[2] of the Federal Reserve Act to lend to non-banks. These emergency measures were followed by a more systematic program that extended to financial and non-financial institutions, and to a limited extent, American households,

2 For section 13, Powers of Federal Reserve Banks, see https://www.federalreserve.gov/aboutthefed/section13.htm.

Table 1
**Government's response to the financial crisis: Relief, recovery, reform**

| Relief and recovery | Reform |
|---|---|
| The Housing and Economic Recovery Act, 2008<br>The Emergency Economic Stabilization Act, 2008<br>The Economic Stimulus Act, 2008<br>The American Recovery and Reinvestment Act, 2009 | The Dodd-Frank Wall Street Reform and Consumer Protection Act, 2010 |

Source: Author's compilation.

Figure 2
**Distribution of TARP funds**
% of total outlay

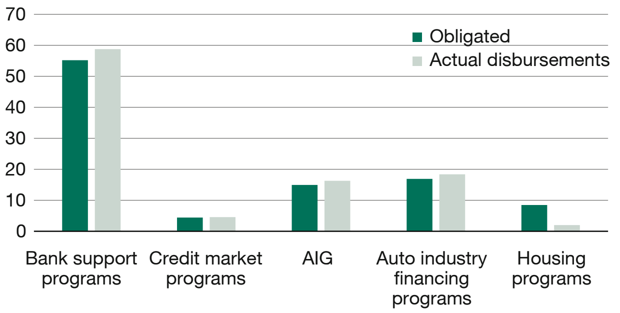

Source: Author's calculation based on data as of June 2013 from B. Wbel (2013), Troubled Asset Relief Program: Implementation and Status, Congressional Research Service.

as US Congress passed a series of Acts aimed at relief, recovery and reform (see Table 1).

## Relief and recovery attempts

*The bailout*

What amount of relief would be provided, to whom, and under what conditions remained a highly contested area of post-crisis governmental response. This is because government intervention often takes place under resource constraints – even if said constraints are political constructs rather than objective reality – and it happens to be, especially in the context of a crisis, fraught with moral questions as well as electoral calculations.

Early on in the crisis, in the spring of 2008, public opinion was largely opposed to the federal government helping Wall Street. Sixty-one percent of those who participated in the Gallup survey[3] conducted in March said that they did not support the federal government taking steps to help major financial institutions to prevent them from failing. Over time, in the face of a deepening crisis, the American public became somewhat less opposed to a Congressional bailout plan.[4] At the same time, it seemed that the majority of that same public considered it important for the government to impose conditions – such as limits on executive compensation – on corporations that would participate in the plan.[5] At that point in time, public confidence in banks was at an all-time low,[6] and there was concern that the government would let those responsible for the economic downturn off the hook.[7]

The key objective of the Housing and Economic Recovery Act, enacted in July 2008, was to bail out the government-sponsored entities Fannie Mae and Freddie Mac, stabilize the mortgage market and prevent a further drop in home prices. The Emergency Economic Stabilization Act passed a few months later, in October, was broader in its reach. It created the Troubled Asset Relief Program (TARP), authorizing the Treasury to spend \$700 billion[8] in a financial rescue plan. The rescue plan entailed the purchase of troubled assets held by financial institutions, capital injections to banks, loans to the auto industry, assistance to American International Group (AIG), and provisional funds for various housing programs including the Home Affordable Modification Program. Looking at the distribution of the funds in terms of percentage of the total outlay (Figure 2) gives a good sense of how TARP money was spent across the board.

As the data shows, although the government's response to the crisis involved measures to provide relief to homeowners as well, the reach of these programs ended up being quite limited overall. The actual disbursements to housing programs were only about 2% of the total TARP disbursements. By the end of 2010, approximately ten million houses had been foreclosed, and millions of homeowners were under water with negative equity, (Martin and Niedt, 2015). The government has thus received criti-

3 For Gallup poll, see https://news.gallup.com/poll/106114/six-oppose-wall-street-bailouts.aspx.
4 See numerous polls conducted in September 2008 by Pew, Gallup and ABC/Washington: https://www.pewresearch.org/2008/10/01/the-bad-rap-on-the-bailout-bill/.
5 https://news.gallup.com/poll/110746/americans-favor-congressional-action-crisis.aspx.
6 Whereas, according to a Gallup survey, 60% of the public indicated a great deal/quite a lot of confidence in banks in 1979, by the time of the bailout, this figure was slightly over 20%. See https://news.gallup.com/poll/183749/confidence-banks-low-rising.aspx.
7 See Pew Research Center survey: https://www.people-press.org/2008/09/30/small-plurality-backs-bailout-plan/.
8 Congress authorized \$700 billion for TARP in October 2008. The Dodd-Frank Act reduced it to \$475 billion. See https://www.treasury.gov/initiatives/financial-stability/TARP-Programs/Pages/default.aspx.

cism that it bailed out the failing financial institutions, but did not do nearly enough for homeowners. This is how a senior Treasury official who served as a TARP administrator explains the government's perspective at the time:

> The most common question I am asked about housing and the financial crisis is why didn't we do more for home owners? Couldn't we have just taken the 700 billion dollars and instead of focusing on banks, just focus on homeowners? Wouldn't that have solved the crisis? My reaction is, the US economy had a massive heart attack. And why does a heart attack kill the patient? A heart attack kills the patient because it deprives blood to their critical organs. And if you have a heart attack, heaven forbid, you go to the emergency room, the surgeon is not going to operate on all your organs. The surgeon is going to go right for the heart, to stabilize the heart, to get blood flowing to the organs. And that's why the capital programs that we talked about earlier were so focused on getting blood flowing in the US economy again or get credit flowing again. (Kashkari, 2018)

Paulson (2018), while agreeing with the heart attack analogy, deemed the notion that the US government had not helped homeowners inaccurate. He argued that while it was hard to convince Americans of this fact – with the workings of credit markets being so complex and obscure – putting Fannie and Freddie under conservatorship was ultimately for the benefit of the homeowners, since without that measure housing prices would have fallen even further. Geithner, who succeeded Paulson as the Secretary of Treasury in 2009, largely agreed with Paulson's assessment while also conceding that housing programs came later than needed and were not large enough:

> The approach we adopted, and we were trying to do the best with the available tools we had, was to try to make sure we brought mortgage rates down. We kept the housing mortgage market open and functioning, so that house prices would start rising again, people wouldn't suffer a massive additional loss of wealth. (2018)

While it is true that the measures that were taken were helpful to some extent, the "trade-off" narrative around relief that was constructed by key policymakers of the time does not reflect the reality. There is no consensus that rushing to the rescue of the source of credit meant that the government's hands were tied to then help the borrowers. This was a political choice, which was likely affected by the public concern over "undeserving" homeowners getting help. This concern was particularly prevalent among high-income groups and Republican voters

Figure 3

**Do you favor or oppose the federal government taking steps to help prevent people from losing their homes because they cannot pay their mortgage (% oppose)**

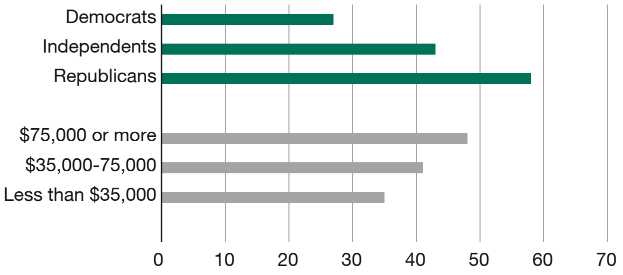

Source: Gallup, https://news.gallup.com/poll/106114/six-oppose-wall-street-bailouts.aspx.

(see Figure 3). The biggest opposition was among Tea Party supporters who were critical of government's relief and recovery attempts in general, and help to homeowners in particular.[9] For policymakers tackling the crisis, therefore, this meant walking a fine line with respect to the "inherent tension between helping lots of people and minimizing waste" (Kashkari, 2018).

*Stimulus packages*

The politics around the stimulus was also contentious. The government attempted to offset the decrease in private spending with an increase in public spending in order to tackle unemployment and stop further economic decline. The key question was how much to spend.

In the US, public discourse around government spending tends to evoke fears of big government and swelling debt. In a national poll conducted in 2011, for instance, only 38% of those surveyed agreed with the statement that "government spending is critical during an economic downturn, even if the government is already running a deficit because government has the unique ability to stimulate the economy through public investment and infrastructure improvement projects that lower unemployment and encourage consumer spending" (Heartland Monitor, 2011, 5). The majority of those surveyed (56%) instead held that "government spending when the government is already running a deficit is the wrong approach during an economic downturn because it is only a temporary solution that increases long-term debt" (Heartland Monitor, 2011, 5). This might explain, to some extent, why

9 See New York Times/CBS News poll: https://www.people-press.org/2008/09/30/small-plurality-backs-bailout-plan/.

the initial stimulus package signed into law on February 13, 2008 by the Republican President George W. Bush – the Economic Stimulus Act – did not go big on spending. Although the Act found bipartisan support, as a stimulus effort, amounting to only 1% of the GDP, it did not have much impact on a $14.4 trillion economy. John Maynard Keynes, in an open letter to FDR had warned the President of the perils of not making recovery through government spending a priority: "During a major downturn only large increases in government expenditures can resuscitate the economy" (1933). In 2008, Keynesian economists voiced similar concerns (see, for instance, Krugman, 2008).

On February 17, 2009, President Obama signed into law the American Recovery and Reinvestment Act (ARRA).[10] At $787 billion, roughly 5.5% of GDP, it was, to quote Christina Romer, then Chair of the Council of Economic Advisors, "the boldest countercyclical fiscal action in American history" (2010). It was also large in comparative terms, as seen in Figure 4. The Act focused on initiatives intended to create jobs and jumpstart the economy, and maintain spending on education and health programs through federal transfers to state governments.

The stimulus package was relatively successful. In 2010, GDP began to grow as unemployment fell. Compared to other financial crises, the length of the Great Recession was shorter and the speed of recovery was faster, as seen in Figures 5 and 6. Despite these relatively favorable figures, many Americans continued to believe that the majority of funds spent under the stimulus bill were "wasted."[11]

## Reform: The Dodd-Frank Act

While the relief and recovery measures had to be put in place immediately, the formulation of regulatory reform took more time and deliberation and was not signed into law until July 2010. The crisis had bared the weaknesses of the regulatory architecture within which the economy operated. The period since the early 1980s had been characterized by the financialization of the American economy. The share of financial institution profits in total corporate profits had increased tremendously, non-financial corporations had begun to invest in financial instruments instead of their core businesses to generate

10 Unlike the Economic Stimulus Act passed earlier, ARRA did not receive bipartisan support.

11 CNN Opinion Research Poll showed that 74% of adults thought that half or more of the funds were wasted. See http://i2.cdn.turner.com/cnn/2010/images/01/25/rel1g.pdf. A more recent poll conducted by the Pew Research Center shows that Americans are still skeptical of stimulus spending, with 41% disapproving of the 2009 stimulus package. See https://www.people-press.org/2012/02/23/auto-bailout-now-backed-stimulus-divisive/.

Figure 4

**Fiscal stimulus in advanced countries**

% of GDP

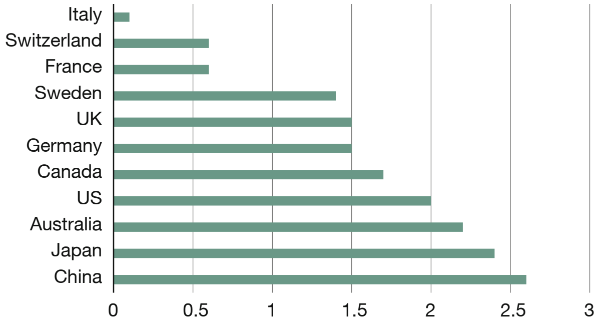

Note: Data captures the magnitude of fiscal stimulus that took effect in 2009 as a share of GDP.

Source: Executive Office of the President of the United States, The Economic Impact of American Recovery and Reinvestment Act.

Figure 5

**Recovery in a comparative perspective: Real GDP change**

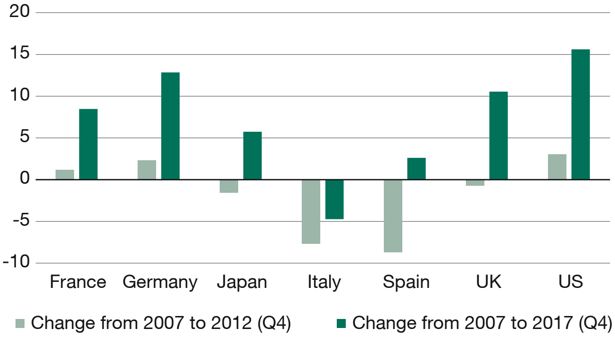

Source: Charting the Financial Crisis: US Strategy and Outcomes, joint report by Hutchins Center on Fiscal and Monetary Policy at Brookings and Yale School of Management Program on Financial Stability.

Figure 6

**Recovery: Great Recession in a historical perspective**

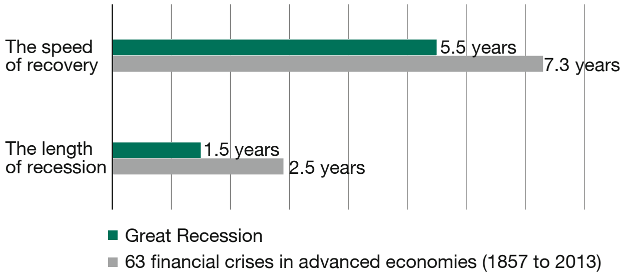

Source: Charting the Financial Crisis: US Strategy and Outcomes, joint report by Hutchins Center on Fiscal and Monetary Policy at Brookings and Yale School of Management Program on Financial Stability.

Figure 7
**Concentration: Assets of five largest banks as a share of total commercial banking assets**
in %

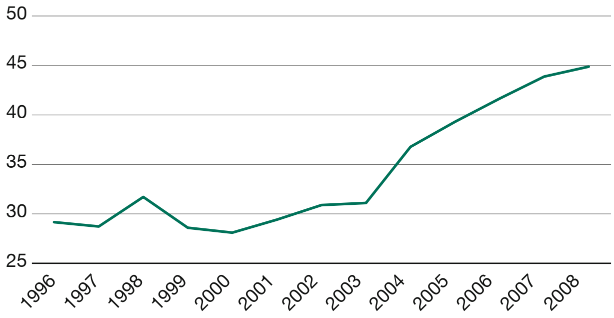

Source: Federal Reserve Bank of St Louis.

revenue and profits, and households and individuals had become more engaged with and dependent on financial markets as borrowers and investors (Krippner, 2011; Fligstein and Shin, 2007).

The change in economic structure was not simply about the increasing size of finance. There had also been significant qualitative changes *within* finance. Non-bank financial institutions had become more prominent actors, the concentration and connectedness of financial institutions had increased tremendously (Figure 7), and complex instruments of structured finance had proliferated, all fundamentally changing the risk formations within and outside the financial sector.

The state's regulatory approach, however, had not kept up with these developments. The regulatory system had remained bank-centered and left the non-bank institutions largely outside of its focus: it had not monitored or mitigated the risk imposed by large and connected institutions; it had not effectively regulated the instruments of structured finance; nor had it put in place updated rules and regulations that would ensure consumer financial protection despite the fact that individuals and households had become increasingly enmeshed in financial markets as borrowers of credit and investors (through their savings or retirement plans). All in all, there was a regulatory deficit in the government's oversight of financial markets.

The Dodd-Frank Act was the government's attempt to deal with this regulatory deficit. The Act created the Financial Stability Oversight Council (FSOC) to monitor the systemic risk, developed a resolution procedure for large financial companies, put in place stricter capital requirements and established the Volcker rule, which prohibited commercial banks from engaging in proprietary trading or investing in hedge funds or private equity funds. It also created the Consumer Financial Protection Bureau (CFPB) tasked with ensuring that consumers will be subject to fair and transparent transactions in relation to loans (mortgages, student loans, credit cards to name a few) and other financial products. The Bureau's jurisdiction extended to non-bank entities such as mortgage brokers and payday lenders.

CFPB was the most contested of the proposed measures. Its establishment was noteworthy for at least two reasons. First, with regard to the history of consumer protection in the US, this is the first time the state has come to fully recognize its responsibility in consumer protection in the financial marketplace. In the past, consumer financial protection responsibility was divided among several agencies, none of which regarded this as its primary objective. Second, the establishment of CFPB represented an expansion of the American regulatory state, and as such ran against the small government direction assumed by the American regulatory policy and discourse over the past 30 years (Vogel, 2012). Despite these important regulatory steps, it has been suggested that Dodd-Frank did not go far enough as a reform attempt in terms of the way it addressed the massive downfall and the causes underlying it. This view seems to have two reference points.

The first one has to do with the size of the downfall. This was not, after all, some little economic downturn. It was a massive crisis, which presented a rather large window of opportunity to change the structure and workings of the financial industry.

The other one is rooted in history: the regulatory reform accomplishments of the New Deal Congress. The US Banking Act of 1933, which had been legislated in the throes of another massive economic downfall, had separated commercial banking from investment banking. Dodd-Frank did not break up the big financial behemoths, achieving little at the end with regard to the too-big-to-fail clause. As such, it is argued (Eichengreen, 2016) that its reach ended up being limited to providing an expansion in regulatory oversight – a set of upgraded rules that would govern essentially the same cosmos of bank and non-bank financial institutions.

While polarization in the legislative environment remained instrumental in the dropping of many substantive, far-reaching proposals early on in the Congressional deliberations of the bill, public opinion itself did not seem

Figure 8
**Public opinion on regulation of business/industry**

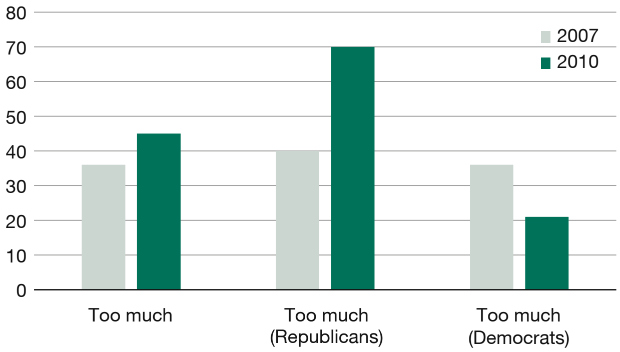

Source: Gallup, https://news.gallup.com/poll/157646/little-appetite-gov-regulation-business.aspx.

to be in favor of a more radical regulatory overhaul. Despite the financial crisis having bared the weaknesses of the regulatory architecture within which finance operated, from 2007 to 2010 there was an increase in the percentage of the populace that thought there was too much regulation – although, it must be noted, this attitude was clearly divided along party lines (see Figure 8).

### The Trump administration

The 2016 presidential election put Donald J. Trump into office, who from the start of his administration, has made the focal point of his policy platrom rolling back the regulatory measures put in place in response to the crisis and, more broadly speaking, limiting the regulatory powers of the government. The Economic Growth, Regulatory Relief and Consumer Protection Act that he signed into law on May 24, 2018 did not quite dismantle Dodd-Frank, as some have argued; however, it rolled back some of its key features and loosened the regulatory oversight over financial institutions in several ways. Dodd-Frank had designated banks with more than $50 billion in assets as systemically important financial institutions and subjected them to higher prudential regulation and capital requirements. The new Act raised the asset threshold for recovery and resolution planning from $50 billion to $250 billion, leaving it to Fed's discretion to apply enhanced regulatory standards to financial institutions with assets between $100 billion and $250 billion. The new Act also exempted banks and credit unions with less than $10 billion in assets from the Volcker Rule.

The Trump administration has arguably shrunk the regulatory oversight of FSOC as well. Under Dodd-Frank, FSOC was authorized to determine whether "a nonbank financial company's material financial distress – or the nature, scope, size, scale, concentration, interconnectedness, or mix of its activities – could pose a threat to U.S. financial stability" (U.S. Department of the Treasury, 2020). Companies designated as systemically important by the Council would be subject to "consolidated supervision by the Federal Reserve and enhanced prudential standards" (U.S. Department of the Treasury, 2020). The Trump Administration moved away from an "institutions-based approach" largely focused on size and connectedness (too big to fail) toward an "activity-based approach," and left it to the primary prudential regulators of those institutions to identify and limit risky activities, essentially defanging FSOC.

The Trump administration also weakened the CFPB. According to a report prepared by the Consumer Federation of America, law enforcement activity at the CFPB has dropped significantly since 2017 (see Table 2). The administration has also challenged the constitutionality of the agency and asked the Supreme Court to limit its independence.

### Ten years after the Dodd-Frank Act

The Great Recession revealed the weaknesses of the regulatory architecture within which America's financialized economy had been operating, particularly with respect to systemic risk mitigation and consumer financial protection. It also revealed the weakness of America's social safety net architecture. Millions of Americans lost their jobs, savings and homes, and found the government to be of little recourse to their woes. A decade later, the American economy bears less risk with respect to some of the issues that contributed to the crisis, while it has become more vulnerable in some other ways.

On the one hand, after the regulatory changes made in response to the crisis, banks' capital and liquidity buffers have increased. On the other hand, concentration in the banking sector has not declined, but in fact has slightly increased.[12] Household debt (including mortgage, credit cards, auto loans and student loans) has been increasing since 2013 and is now above the 2008 level. Corporate debt has also been increasing and has reached three-fourths of GDP, which is also higher than it was in 2008. Non-bank financial institutions pose some concerns as well. Several large institutions that were deemed systemically important by the FSOC in the aftermath of the crisis were relieved of that designation, hence enjoying regulatory relief. The weakening of the FSOC is a concern

12 See https://fred.stlouisfed.org/series/DDOI06USA156NWDB, 5-bank asset concentration.

Table 2
**Enforcement at Consumer Financial Protection Bureau**

| | | Directors | |
|---|---|---|---|
| Restitution | Cordray | Mulvaney | Kraninger |
| Enforcing the equal credit opportunity act | | | |
| Number of cases | 11 | 0 | 0 |
| Total consumer relief | $618,726,890 | 0 | 0 |
| Student lending | | | |
| Number of cases | 15 | 0 | 0 |
| Total consumer relief | $712,530,184 | 0 | 0 |
| Enforcing fair credit reporting | | | |
| Number of cases | 24 | 2 | 0 |
| Total consumer relief | $390,157,992 | 0 | 0 |
| Enforcing mortgage-related cases | | | |
| Number of cases | 61 | 2 | 0 |
| Total consumer relief | $2,969,543,550 | $268,869 | 0 |
| All cases | | | |
| Number of cases | 201 | 11 | 5 |
| Total consumer relief | $11,980,130,720 | $345,094,707 | $12,028,522 |

Note: The figure for "all cases" includes categories that are not on this table.

Source: Peterson, 2019.

given that the Council was supposed to watch for and mitigate systemic risk in relation to this sector, whose liquidity level remains lower than that of banks. All of this is all the more concerning given that the prominence of non-bank financial institutions in mortgage markets has increased since the Great Recession.

In sum, the American economy still embodies significant structural vulnerabilities ten years after the end of the Great Recession, which had drastic consequences for the well-being of millions of Americans. There seems to be concensus among political observers that the socio-economic vulnerabilities that the Great Recession brought to fore were instrumental in making room, politically speaking, for populist discourses, and determining the course of the 2016 elections (Judis, 2016).[13]

13 Also see https://www.gsb.stanford.edu/insights/how-great-recession-influenced-todays-populist-movements.

## From the Great Recession to the Great Lockdown

Presently, the US is heading into the 2020 presidential election facing yet again an economic downturn and uncertainty, this time fueled by a pandemic. The IMF (2020) dubbed the slowdown in economic activity resulting from the public health measures that have been taken to combat the coronavirus – such as quarantines and business closings – the Great Lockdown. Contact-intensive industries have been experiencing a drop in the demand for their products and services. These industries employ about one-third of the American workforce,[14] and therefore it is not surprising that unemployment rose sharply in March and April of 2020. Currently, as of June, the unemployment rate is above 11%, and the four-week moving average of continued unemployment benefit claims is above 20 million.[15] According to the recent projections of the Federal Reserve,[16] the economy is likely to contract by 6.5% by the end of 2020.

It is not surprising that in this juncture, questions pertaining to the government's role in monitoring and managing risks, mitigating socio-economic vulnerabilities and boosting the economy have become salient, once again, subject to electoral calculations, moral considerations and institutional limitations. While the Great Lockdown is fundamentally different from the Great Recession in terms of its nature and causes, it poses similar questions in relation to the government's role in providing relief, helping the economy recover and in monitoring, identifying and mitigating systemic risks.

With respect to relief and recovery, the US government has undertaken a number of steps so far. On March 6, the Coronavirus Preparedness and Response Supplemental Appropriations Act was signed into law. This was followed, on March 18, by the Families First Coronavirus Response Act; on March 27, the CARES Act – the largest stimulus package in US history – and on April 24, Paycheck Protection Program and Health Care Enhancement Act. Together, these governmental interventions provided a number of relief measures including a one-time individual tax rebate, corporate tax relief, expansion of unemployment benefits, small businesses assistance and transfers to state and local governments. On the monetary side, the Federal Reserve has also taken a number of decisive steps to provide liquidity to markets and prevent a credit crisis. Reaching recovery is more

14 See data from Federal Reserve Bank of St Louis, https://www.stlouisfed.org/on-the-economy/2020/april/impact-social-distancing-ripples-economy.
15 As of June 6. Please see https://fred.stlouisfed.org/series/CCSA.
16 See https://www.federalreserve.gov/monetarypolicy/fomcprojtabl20200610.htm.

challenging in this case, relative to the Great Recession. Back then, the task at hand was to increase consumer spending and get people back to work, whereas now some sectors of the economy need to remain closed due to public health concerns and cannot be resuscitated with stimulus spending.

As for relief, the limitations of American social welfare policy have once again been laid bare. The one-time $1200 individual relief checks provided to those eligible are in no way an adequate compensation for the loss of income and benefits that millions of Americans have experienced. When compared with the various employment protection, wage replacement and paid leave programs that are available in European countries, the degree to which American capitalism operates devoid of any substantial safety net becomes obvious.

What is more telling perhaps is the failure of the government to monitor and mitigate systemic risks, for that is what COVID-19 constitutes: a systemic risk. Its spread is due to a multitude of interrelated global causes, its impact is broad and cannot be localized, and combating it requires an integrated and multifaceted approach. The inability of the government to monitor and identify such a large-scale risk to public health, which would inevitably have massive economic consequences, speaks to the shortcomings of the American government as a risk manager, which we had also seen in the context of the Great Recession.

In short, as different as they are, both crises have revealed the limitations of American statecraft. In the aftermath of the Great Recession, those limitations have played a role in the public's turn to anti-establishment politics. This time around, the crisis might inspire a different political response, especially because its toll is evident not only in terms of negative growth rates and rising unemployment, but also in lives lost.
